# Study on the Influence of Delamination Damage on the Processing Quality of Composite Laminates

**DOI:** 10.3390/ma15238572

**Published:** 2022-12-01

**Authors:** Jiali Yu, Yimeng Shan, Yiming Zhao, Ran Mo

**Affiliations:** 1 State-Owned Wuhu Machinery Factory, Wuhu 241000, China; 2 Science and Technology on Thermostructural Composite Materials Laboratory, Northwestern Polytechnical University, Xi’an 710072, China

**Keywords:** composite material, delamination damage, axial force, processing quality

## Abstract

Internal delamination damage in composite connection structures can occur in the process of the overloading of a high-speed bearing, with alternating force loads, high or low temperatures, and the humid or hot environment loads. Mechanical drilling and riveting are usually used at the delamination position and outside its envelope, to inhibit delamination expansion. However, delamination damage can change the structural stress state of the original structure. It is difficult to achieve a better inhibition effect using conventional drilling mechanisms and process methods with intact composite panels, and new damage forms can even be introduced into the drilling process due to unreasonable parameter settings. Therefore, this paper combined finite element simulation technology and experimental processing technology, to analyze the influence of different delamination dimensions and positions on processing quality. The results showed that the feed speed and rotating speed had significant effects on the axial force of composite laminates. In particular, in the case of a low speed and high feed, the axial force will increase significantly.

## 1. Introduction

Fiber-reinforced composites have the advantages of high specific strength, high specific stiffness, and good fatigue resistance and design-ability, and are gradually being implemented in aerospace, shipping, rail transit, and other fields. Their applications are also gradually transitioning from secondary load-bearing components, to main load-bearing components, such as fuselage wallboard, hull wallboard, etc. [1,2,3].

The macro-mechanical properties of composites are anisotropic, and the strength in each direction is also very different, leading to the interlayers being the weakest position of composites. During the preparation of composite panels, due to the variation of laying quality, resin rich areas can result in differences in the shrinkage between layers during curing, as well as delamination defects [4]. At the same time, incomplete curing of materials, entry of foreign particles, and uneven heating of equipment can also cause interlayer debonding, which results in initial interlaminar damage or cracks at the root of composite panels at the beginning of service. In addition, when an aircraft is impacted and damaged by foreign objects; in alternating environments of high temperature, low temperature, and high humidity, not only the original initial interlayer defects and damages further expand under these extreme conditions, but also new delamination damage can form and expand due to overloading [5,6,7,8].

For the composite delamination damage induced by material formation or structural service, mechanical connection technology is usually used; that is, drilling and riveting in the delamination area, to inhibit the initiation and propagation of interlayer cracks [9]. However, unlike sound composites with an extremely strong cohesive adhesion between layers, composite structures with delamination damage are prone to serious secondary damage during drilling and riveting, which seriously affects the repair strength and fatigue life of the connecting structures [10,11,12,13]. Therefore, many scholars have also carried out relevant research on drilling mechanisms, and the process and assembly of composite panel structures.

In fact, delamination defects change the distribution of interlaminar stress in composites and the bearing capacity of components [14,15]. The conclusions of the initial drilling damage research based on intact composite structures can no longer be used as a reference and applied to composites with delamination damage points [16,17,18,19,20]. In combination with ultrasonic C-scans, acoustic emission, virtual crack closure, and other technical means, researchers have conducted in-depth research on out of plane displacement, hole circumference stress delamination buckling, and the failure load of composite materials with delamination damage [21,22,23,24]. This research showed that with the increase of delamination size, the critical buckling load decreases. The critical buckling load of a circular layered structure is smaller than that of a rectangular layered structure. The delamination propagation path first extends along the width direction to the center, and then along the length direction [25,26]. Further research showed that the damage is layer-dependent in a laminate, and different fiber materials influence the delamination resistance in different ways [27,28,29]. The closer the delamination is to the surface, the lower the bearing capacity of the specimen, and the easier it is for interface cracking, interlaminar cracking, and fiber fracture to occur. When laminated plates with multiple delamination defects are subjected to a compression load, the buckling and post-buckling of the sub-plates of laminated plates cause delamination expansion, and this delamination expansion also affects the post-buckling of sub-plates. The interaction between them significantly decreases the residual compressive strength of the laminated plates [30,31,32,33].

Although researchers have studied the damage propagation law of composites with delamination defects from the perspectives of defect geometry, mechanical properties, and failure prediction, the existing experimental and numerical analyses mainly focused on static loads, such as bending and compression [34,35]. In the actual drilling process, the drilling is a dynamic process under continuous tool feeding; therefore, the delamination propagation law under a static load cannot fully describe the stress state distribution and damage propagation path in the delamination area during the dynamic drilling process of composites. There is no clear damage mechanism and design method for processing parameters for composite processing with delamination damage. At present, the processing parameters used are the same as those for intact composite samples, thus lacking pertinence and effectiveness.

Therefore, this paper used a composite panel structure with initial delamination defects as research object, and the drilling process and connection technology were studied. Through orthogonal testing and finite element simulation analysis of the different drilling parameters of composites, the damage expansion and inhibition mechanism of composites under a dynamic load were explored, which provided a basis for drilling defect control and variable parameter drilling process optimization.

## 2. Experiment

### 2.1. Sample

**Figure 1:**
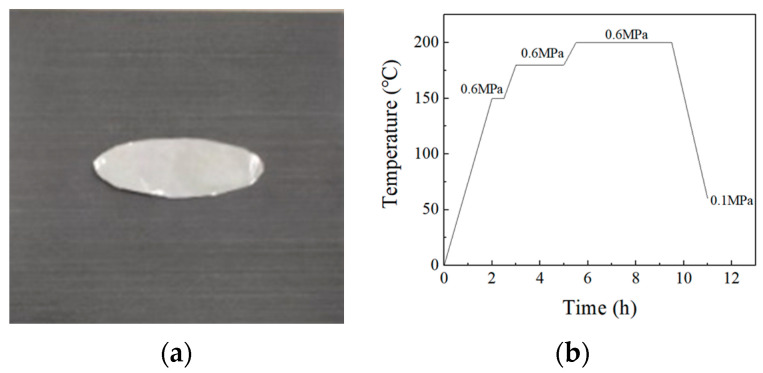
(**a**): Aluminum foil, (**b**): Composite molding curve.

The test material in the paper was a carbon fiber reinforced resin matrix composite (ZT7H/5429) unidirectional belt prepreg purchased from AVIC Composite Co., Ltd. (Beijing, China). First, according to the paving sequence of [45/−45/0/−45/45/0/−45/90/45/−45/−45/0]s from the wing skin of a certain type of aircraft, an automatic blanking machine was applied to cut the prepreg, where the 0° direction was the fiber direction. Then, an oval aluminum foil with a 90 mm long axis and 30 mm short axis was cut. During the laying of the laminate, the foil was placed at 3/4 of the thickness of the laminate, to prefabricate delamination defects, and to make 1/4 of the thickness of the laminate as a comparison sample, as shown in Figure 1. The laminates were cured under high temperature and pressure after laying, and the curing curve is shown, as follows. Finally, the edges were trimmed to obtain the center area with the best curing quality.

### 2.2. Drilling Test

An XK7124 CNC vertical bed drilling machine manufactured by Zoje CNC Machine Tool Co., Ltd. (Yuhuan, China). was used for the drilling processing test. The force measuring instrument used in the test was a 9129AA multi-component force measuring instrument manufactured by the Kistler Company. The test processing tool was a 4.05 mm carbide steel drill bit manufactured by the Sandvik Company. According to the test design matrix of the drilling parameters, each group of drilling processing samples should contain at least 3 samples.

In order to simulate the stress state of the in situ drilling processing of composite wall panels, the unsupported drilling mode was adopted in this test and no base plate was used. In order to avoid damage to the dynamometer when the drill bit drilled out the workpiece, a support block was placed between the workpiece and the dynamometer, to ensure that the drill bit had enough cutting space.

**Figure 2:**
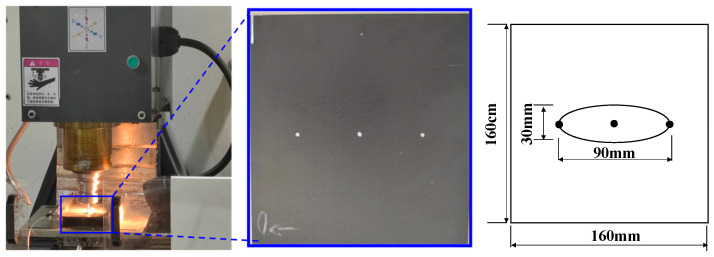
Test equipment and sample.

Before the test, a force measuring instrument was fixed on the workbench of the vertical machine tool, and the carbon fiber composite laminate was tightened and fixed on the force measuring instrument using hexagonal socket bolts. When the force measuring instrument was activated, the force generated during the drilling process was converted into an electrical signal value of the same size using a charge amplifier. The data acquisition card collected these electrical signals, and then converted them into a digital signal that could be recognized by the computer via an A/D converter. Finally, corresponding software on the computer was used to collect and analyze the data. By controlling the processing machine tool, 3 holes were drilled on the delamination and envelope line of the sample, as shown in Figure 2. And each experiment was repeated 3 times.

### 2.3. Finite Element Simulation Model

**Figure 3:**
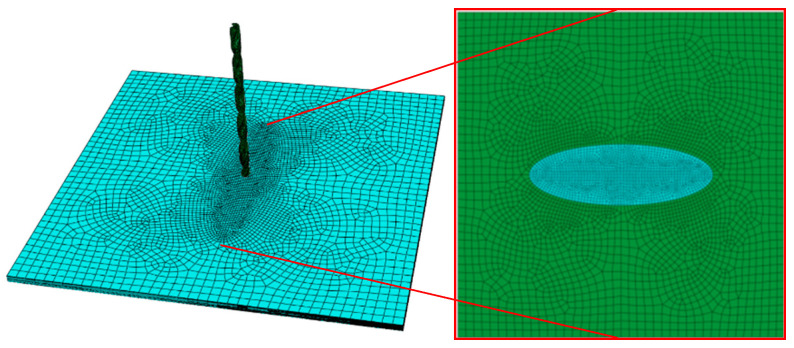
Finite element model and the grid cell.

With the application of ABAQUS 6.14 finite element analysis software, an overall model, including the laminated composite plate with layers and drill bit, was established, and the paving angle was consistent with the sample. HyperMesh was applied to divide and mesh the drill bit model. A hexahedral mesh was used as the mesh, and the mesh size was 0.5 mm; the overall seed size of the laminated plate grid was 4 mm, the seed size of the prefabricated layered outer boundary was 2 mm, and the seed size of the layered central boundary was 1 mm. Then, a layer of cohesive grid cells with a thickness of 0 was established using grid offset, and the center was 90 mm. The mesh deletion of an 30 mm elliptical area achieved the purpose of pre-layering. The model and grid results are shown in Figure 3.

Regarding the material property settings, the drill bit was made of high-speed steel, with an elastic modulus of 200 GPa, Poisson’s ratio of 0.3, and a density of 6.7 g/cm^3^. The material parameters of the ZT7H/5429 composite laminate are shown in Table 1.

When setting the interaction and load boundary, the drill bit was set as a rigid body, and the contact friction coefficient between the drill bit and the laminated plate was set as 0.3, considering that the drill bit has almost no deformation with respect to the laminated plate in the actual process. The four end faces of the laminated plate were set with full constraints, and the translational and rotational degrees of freedom along the three main directions were constrained. A 3000 rpm speed and 5 mm/min feed speed were applied with the drill control point. Considering that the composite material processing is a dynamic process, this model used a Dynamic Explicit solver and a 1e6 mass scaling factor to speed up the calculation.

## 3. Results and Analysis

### 3.1. Analysis of the Drilling Damage Propagation Mechanism

**Figure 4:**
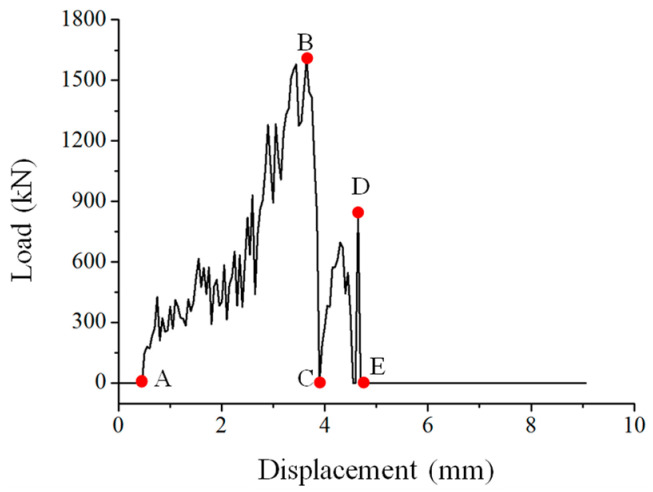
Drill axial force–displacement curve. A: Machining starting point; B: Ultimate load point; C: Secondary damage starting point; D: Ultimate secondary damage point; E: Machining end point.

For the finite element calculation results, the axial force of the drill along the feed direction (Z direction) was extracted. It was found that the axial cutting force of the drill changed with the drilling process. As can be seen from Figure 4, the processing stage can be divided into six stages.

At the O-A contact stage, the drill bit and the laminate began to be in contact, and the reaction force in the Z direction was zero. At this time, the laminate only had processing stress at the center, which was only 4.07 MPa. There was no damage to the laminate, and there was no change in the cohesive.

**Figure 5:**
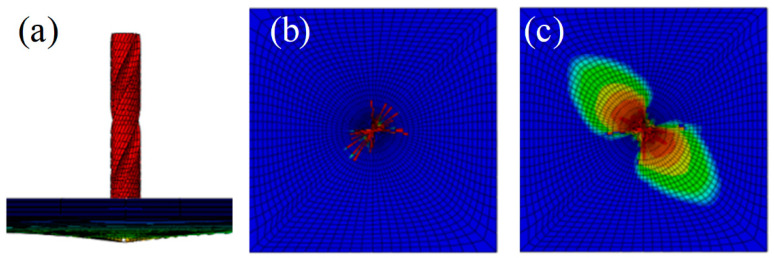
Damage during the feeding stage. (**a**) Sag deformation, (**b**) matrix compression failure, (**c**) fiber compression failure.

The drill bit in the A-B feeding stage gradually contacted the laminated plate, and the drill blade cut the laminated plate. At the same time, under longitudinal feeding of the drill bit, the laminates gradually sagged and deformed, and damage was initiated, mainly due to compression. Since the top layer of the laminate was 45° ply, the fiber damage also showed obvious directionality. The continuous feeding of the drill bit caused the circumference of the prefabricated delamination to stretch outward, and slight delamination expansion appeared around this point, as shown in Figure 5.

**Figure 6:**
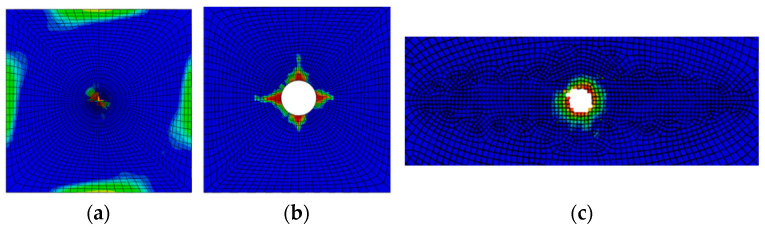
Damage around the hole. (**a**) Fiber compression failure; (**b**) initial delamination damage propagation. (**c**): Initial delamination damage after penetration.

At the rebound stage of B-C, the laminated plate above the layer had been penetrated by the drill bit, and the laminated plate started to rebound, with the maximum downward depression 1.2 mm smaller than that of point B. At this stage, the hole wall material and the drill bit contact increased, the fiber and matrix were obviously damaged, and the compression and tensile damage were significantly expanded. It is worth noting that the fiber damage at the outlet of the lower layer of the laminated plate was selected at this stage, and a certain amount of fiber damage (as shown in Figure 6a,b) occurred around the laminated plate. This was because the boundary conditions around the boundary could not be free and automatic, and damage occurred due to the existence of internal stress. In practical engineering structures, such as aircraft and automobiles, similar damages are often more obvious. This is because multiple composite materials tend to accumulate assembly stress during the assembly process. In the processing process, drilling leads to local stress release.

In the C-D secondary feeding stage, each layer in the lower part of the laminated plate continued to move downward under the action of the drill bit, and the maximum displacement was 0.4 mm larger than that in the B-C stage. However, compared with section A-B, the overall thickness and stiffness of the laminate were reduced, and a certain amount of fiber and matrix damage had been accumulated in the early stage, so this process was much faster than that of section A-B. Since the drill bit is always fed along the thickness direction, how to optimize the processing parameters according to the ratio of the thickness of the processed material and the unprocessed material during processing is the key to improving the processing quality

In the D-E penetration stage, the drill bit broke through the entire laminated plate at moment E, and then the damage around the outlet gradually increased. As shown in Figure 6c, the axial force in the Z direction at the tip of the drill bit was always zero, and the entire drilling processing stage also ended. At this stage, if the processing parameters of the drill are be well adjusted, the damage near the outlet increases sharply.

It can be seen from the finite element simulation analysis that, during the mechanical drilling process, the main reason for the increased damage to the composite laminates was the existence of delamination damage in the middle of the book, which led to secondary feeding of the drill bit on the laminates, and especially serious damage to the laminates below the delamination. In order to ensure the quality of processing, it was necessary to control and design the processing parameters at different test stages.

### 3.2. Analysis of Machining Axial Force

By monitoring the change process of the axial force of the drill bit with time during the drilling process, the influence of the drill bit speed and feed speed on the axial force of the drilling process were analyzed under a steady state. The specific test design matrix is shown in Table 2.

The sampling rate of the dynamometer in this work was 10,000 Hz. In order to improve the efficiency of data processing, Origin was used for data processing. First, FFT filtering was performed, with a filtering interval of 70. Second, the order calculation method was adopted, and a 100:1 ratio was adopted to reduce the data density.

#### 3.2.1. Effect of Layer Thickness

**Figure 7:**
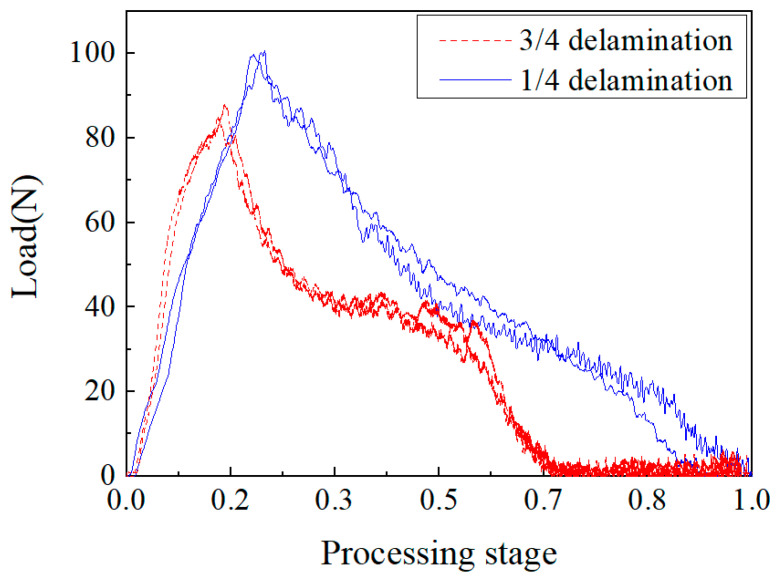
Axial force–time curve at different delamination positions.

Figure 7 shows the change curve of the drill axial force with time for composite laminate samples with a 1/4 delamination and 3/4 delamination during drilling, at the same speed and feed rate, and compares the influence of different delamination positions on the cutting force. It can be seen from the figure that there were differences in the characteristics of the processing load curves at different delamination positions. This was due to the different contact distances with the drill bit, which made the secondary feeding stage of 1/4 delamination sample occur earlier than that of the 3/4 delamination sample. The maximum axial cutting force of the two samples was also different. The maximum cutting force of the 1/4 layered sample was 86.4 N, and the maximum cutting force of the 3/4 sample was 100.2 N, which is about 15.8% higher. This was because, at the 1/4 delamination, the interface was open, and there was still a 3/4 thick laminated plate under the drill bit, which required a greater axial force for feeding.

#### 3.2.2. Effect of Feed Speed

**Figure 8:**
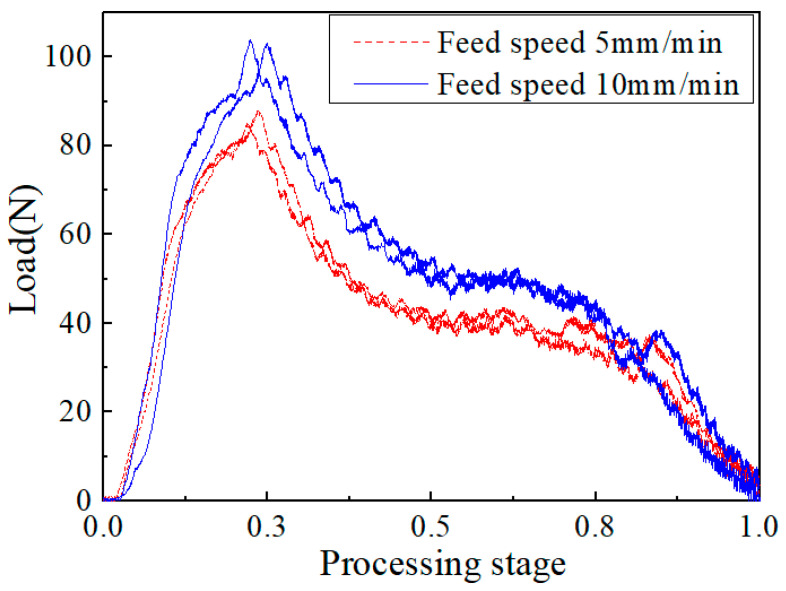
Axial force–time curve at different feed speeds.

Figure 8 shows the change curve of the axial force of the composite laminate sample with 3/4 delamination during drilling, at the same speed of 3000 rpm with two feed speeds, and shows the influence of different feed speeds on the axial cutting force. It can be seen from the figure that, although the characteristics of the machining load curves at different feed speeds were basically the same, the maximum cutting force of the 5 mm/min sample was 86.4 N, while the maximum cutting force of the 10 mm/min sample was 103.6 N, which is about 20% higher. This shows that the feed speed is the main factor affecting the drilling force. When the feed speed increases, the drill needs to cut a greater thickness of layers in one unit of time, the corresponding maximum cutting force is higher, and the impact on the drilling quality is greater.

#### 3.2.3. Effect of Rotation Speed

**Figure 9:**
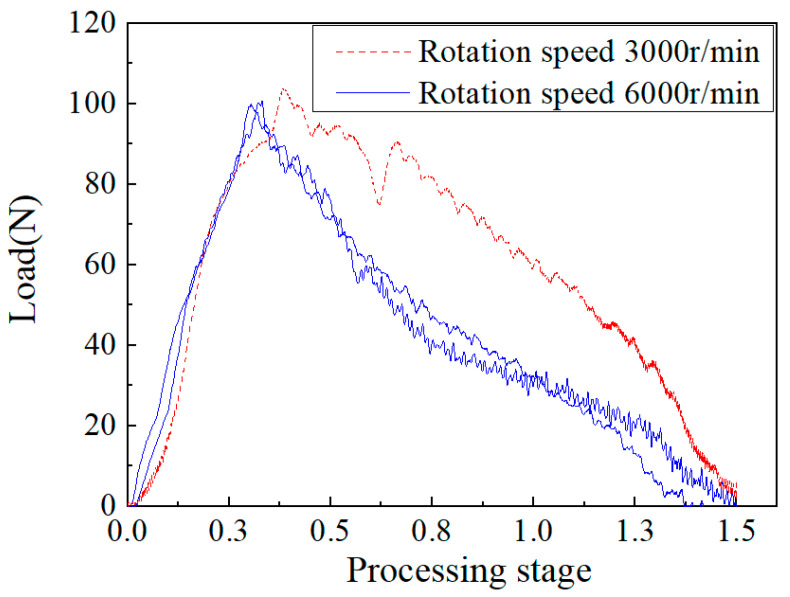
Axial force–time curve at different speeds.

Figure 9 shows the change curve of the axial force of the composite laminate sample with 3/4 delamination during drilling at the same feed speed of 5 mm/min under two rotating speeds, and discusses the influence of different rotating speeds on the axial cutting force. It can be seen from the figure that the load displacement curves of the machined samples at different speeds were relatively close, and the maximum cutting forces were 103.9 N and 100.3 N, respectively. These values are relatively close, with a difference of nearly 3.6%, indicating that the speed difference had relatively little influence on the maximum cutting force.

In general, the feed speed and delamination thickness had the greatest influence on the machining axial force. Among the factors, the influence of the rotating speed on the cutting force of laminate was similar to that of traditional laminate, and the two were positively correlated. With an increase of the delamination thickness, the materials to be machined by the drill bit after the delamination interface gradually decreased, and the axial drilling force decreased.

## 4. Conclusions

In the paper, a carbon fiber reinforced resin matrix composite laminate with initial delamination was taken as the research object, and the drilling parameters of delamination inhibition and connection technology for the delamination samples were controlled and designed. With the axial force as the judgment index, the influence of different processing parameters on the drilling quality was compared and analyzed, and the following conclusions were drawn:

(1) Based on finite element simulation software, a dynamic machining simulation model of composite laminates with delamination was established. Through analysis of the change in axial force of the drill bit, the damage to the laminates, and the expansion of the original delamination, it was revealed that the damage to composite laminates during the drilling process mainly included three stages: feed compression damage, rebound damage expansion, and secondary feed inner wall delamination expansion.
(2) With the help of vertical CNC machine tools and precision force measuring instruments, a constant parameter drilling test design was carried out for the laminated composite plate samples containing layers, and the effects of different processing parameters on the processing axial force were qualitatively compared. The results showed that the effect of rotational speed on the cutting force for the composite laminates was similar to that of intact composite laminates, and the two were positively correlated. The feed speed and layering position had the greatest influence on the axial force of machining. The faster the feed speed, the greater the axial force of machining. With the increase of layering thickness, the axial force of machining decreased. Similar results were shown in in a drilling experiment of intact composite laminates. The drilling feed speed had a great influence on the drilling axial force. When the drilling feed speed was higher, the drilling axial force is larger; the delamination factor also increased, and the delamination defect was aggravated [36].

## Figures and Tables

**Table 1:** Performance Parameters of the ZT7H/5429 Composite.

E_1_ (GPa)	E_2_ (GPa)	G_12_ (GPa)	G_13_ (GPa)	X_T_ (MPa)
140	9	5	5	1400
Y_C_ (MPa)	Y_T_ (MPa)	X_C_ (MPa)	S_12_ (MPa)	S_23_ (MPa)
50	50	180	99	99

**Table 2:** Constant parameter drilling design matrix.

Serial	Rotation Speed (r/min)	Feed Speed (mm/min)
**1**	3000	5
**2**	3000	10
**3**	6000	5
**4**	6000	10
